# Book Reviews

**Published:** 1900-02

**Authors:** 


PROGRESSIVE MEDICINE. Volume IV. A Quarterly Digest of Advances, Discoveries and Improvements in the Medical and Surgical Sciences. Edited by HOBART AMORY HARE, M. D., Professor of Therapeutics and Materia Medica in the Jefferson Medical College of Philadelphia. Octavo, handsomely bound in cloth, 398 pages, fifty-one engravings and five plates. Philadelphia and New York: Lea Brothers & Co.

This volume completes the first year of this useful digest. It will be found considering, first, diseases of the digestive tract and allied organs, the liver, pancreas and peritoneum, by Charles G. Stockton, of Buffalo. Second, genito-urinary diseases in the male, and syphilis, by William T. Belfield, of Chicago Third, fractures, dislocations, amputations, surgery of the extremities, and orthopedics, by Joseph E. Bloodgood, of Baltimore. Fourth, diseases of the kidneys, by John Rose Bradford, of London. Fifth, physiology, by Albert P. Brubaker, of Philadelphia. Sixth, anatomy, by Frederic H. Gerrish, of Portland, Me. Seventh, hygiene by Henry B. Baker, of Lansing. Eighth, practical therapeutic referendum, by E. Q. Thornton, of Philadelphia.

It will be observed that these contributors are, for the most part, men well known in medicine, and, as therefore might be expected, these articles are the latest and best expressions of the art and science of medicine and surgery up to the end of the year 1899, to which the volume takes us. We have heretofore expressed our opinion of "Progressive Medicine," hence need not repeat it here, except to say that as each volume has appeared, and especially in view of this last one, we are more and more strengthened in the belief that it is a great aid to the over-worked physician who must needs economise his time even to the moments.

---

Coakley on the Nose and Throat. The Diagnosis and Treatment of Diseases of the Nose, Throat, Naso-Pharynx and Trachea. For the Use of Students and Practitioners. By Cornelius G. Coakley, M. D., Professor of Laryngology in the University and Bellevue Hospital Medical College, New York. In one handsome 12mo volume of 536 pages, with 92 engravings and 2 colored plates. Philadelphia and New York: Lea Brothers & Co. 1899.

Almost every branch of medicine is supplied with a manual. In this one a specialty, heretofore without literature of this order, is now provided. The book is intended for both students and practitioners, hence will undoubtedly fill the place to which we have referred and we may as well say right here that, in our opinion, it does it well.

The practical side of the subject is amply discussed, the chapters on diagnosis and treatment having received special attention at the author's hands. Chronic hypertrophic tonsillitis is well handled, which is a very important condition, and one that should be relieved before it leads to complications that may prove intractable. Diphtheria receives due consideration, which is another disease that admits of no delay in treatment. Disease of the maxillary sinus, sometimes so obscure, is receiving more attention of late by specialists, and we are pleased to see it so satisfactorily dealth with by Coakley. Tuberculosis of the larynx, too, a disease that must be recognised early in order to justify expectancy of cure, is well considered in this book. A considerable space is taken up with the subject of foreign bodies in the upper air tract, which is one of practical importance.

There are many other topics that deserve special mention in this book, but perhaps we have said enough to serve our purpose, which has been to accentuate the fact that this is one of the most satisfactory manuals that has recently come to our attention. It is well illustrated and all through the book are indications that it is the product of brain and hands that are familiar with the needs of the worker in the field that it deals with.

---

The Treatment of Pelvic Inflammations Through the Vagina. By William H. Pryor, M. D., Professor of Gynecology, New York Polyclinic; Consulting Surgeon, City (Charity) Hospital, etc. 12mo, pp. 248. With 110 illustrations. Philadelphia: W. B. Saunders. 1899.

This interesting monograph details the work of one of the most capable of the advocates of the vaginal route in preference to the abdominal, for most operations within the pelvic cavity. He gives his reasons for this predeliction with potency and records them in unmistakable English.

Beginning with endometritis, which is discussed under its several forms in about 35 pages, he proceeds to pelvic inflammation proper (9 pp.), salpingitis, under several heads (27 pp.), pelvic peritonitis (14 pp.), tubercular peritonitis (2 pp.), inflammatory diseases of the ovaries (8 pp.), broad ligament cyst (3 pp.), adherent retropositions (4 pp.), broad ligament abscess (2 pp.), diffuse pelvic suppuration (2 pp.), the anesthetic (1pp.), curettage (10 pp.), exploratory vaginal section (10 pp.), conservative treatment (5), preparation of patient for a vaginal section (2 pp.), vaginal ablation (46 pp.) morcelation (6 pp.), vagino-abdominal hysterectomy in the puerperal state (4 pp.), accidents and complications (4 pp.), secondary hemorrhage (2 pp.), intravenous injection of normal salt solution (2 pp.), instruments (6 pp.), formulæ (1 pp.), sterilisation (3 pp.), index (8). There are several well-drawn illustrations in the book taken from life, and a number of others that are equally useful in explaining the author's method or ideas.

It is a book that will please the aggressive operator by the vaginal route and all who think with him that his way is the best.

---

A Compend of the Practice of Medicine. By Daniel E. Hughes, M. D., Chief Resident Physician Philadelphia Hospital; Physician-in-Chief Insane Department, Philadelphia Hospital, etc. Sixth Physicians' edition, thoroughly revised and enlarged, including a section on mental diseases and a very complete section on skin diseases. Philadelphia: P. Blakiston's Son & Co. 1899.

This excellent compend is again presented to the profession of medicine in a most attractive form. It is printed on excellent paper, with clear faced type, in gilt edges, with rounded corners and bound in flexible morocco.

The contents of the book, most of which is of the best material, is so arranged as to be readily accessible. It is, moreover, of convenient size for ready reference, has been prepared by a man who knows what busy physicians need and who has prepared for them just such a work.

It has been in popular demand too long to need more than a mere hint that a new edition is ready, to insure its purchase by those who desire to be fully equipped with the best. The price, $2.25, is so reasonable as to bring it within the reach of the majority. We regard it as one of the most useful compends that is now in the market.

---

Minor Surgery and Bandaging. By Henry R. Wharton, M. D., Demonstrator of Surgery in the University of Pennsylvania. New (4th) edition. In one 12mo volume of 594 pages, with 502 engravings, many being photographic. Philadelphia and New York: Lea Brothers & Co.

The profession of medicine, as well as undergraduates are familiar with this manual. It has received the favorable judgment of teachers, practitioners and students, since it was first published in 1891. At that time the JOURNAL gave it a detailed examination and expressed its opinion with considerable fulness. The succeeding editions have all kept pace with the progress of events, and this one is unusually satisfactory in that direction.

It is a book that exhibits the handiwork of the practical surgeon in all sections. In it, too, are described many little niceties in surgery, if we may so term them, that are rarely found elsewhere, which alone make the book of special value to the beginner in surgery, not to speak of its usefulness in that respect to his more advanced colleague.

A new section has been added detailing operative procedures on the cadaver, which will be found of interest especially in relation to the ligation of bloodvessels. The illustrations are excellent and the work deserves the full measure of success that it has attained.

